# GPUs, a New Tool of Acceleration in CFD: Efficiency and Reliability on Smoothed Particle Hydrodynamics Methods

**DOI:** 10.1371/journal.pone.0020685

**Published:** 2011-06-13

**Authors:** Alejandro C. Crespo, Jose M. Dominguez, Anxo Barreiro, Moncho Gómez-Gesteira, Benedict D. Rogers

**Affiliations:** 1 EPHYSLAB Environmental Physics Laboratory, Universidade de Vigo, Ourense, Spain; 2 School of Mechanical, Aerospace and Civil Engineering, University of Manchester, Manchester, United Kingdom; German Cancer Research Center, Germany

## Abstract

Smoothed Particle Hydrodynamics (SPH) is a numerical method commonly used in Computational Fluid Dynamics (CFD) to simulate complex free-surface flows. Simulations with this mesh-free particle method far exceed the capacity of a single processor. In this paper, as part of a dual-functioning code for either central processing units (CPUs) or Graphics Processor Units (GPUs), a parallelisation using GPUs is presented. The GPU parallelisation technique uses the Compute Unified Device Architecture (CUDA) of nVidia devices. Simulations with more than one million particles on a single GPU card exhibit speedups of up to two orders of magnitude over using a single-core CPU. It is demonstrated that the code achieves different speedups with different CUDA-enabled GPUs. The numerical behaviour of the SPH code is validated with a standard benchmark test case of dam break flow impacting on an obstacle where good agreement with the experimental results is observed. Both the achieved speed-ups and the quantitative agreement with experiments suggest that CUDA-based GPU programming can be used in SPH methods with efficiency and reliability.

## Introduction

In the study of fluid mechanics, computational fluid dynamics (CFD) has become commonplace in industry and academic research to investigate flows of great complexity. The rapid improvement of computational resources has lead to the development and application of a variety of mesh-based techniques including finite elements methods, finite volume and finite difference discretisations. In recent years, numerous meshless methods have appeared and grown in popularity as they can be applied to problems that are highly nonlinear in arbitrarily complex geometries and are difficult for mesh-based methods. Of the meshless techniques now available, smoothed particle hydrodynamics (SPH) is proving popular and robust.

As a Lagrangian method, SPH does not require a computational mesh, and has attracted considerable interest during the last decade in a variety of fields, in particular, the study of free-surface flows. Originally invented for astrophysics during 1970s [1], [2], it has been applied to many different fields of fluid dynamics and solid mechanics. Instead of using a mesh, the SPH method uses a set of interpolation nodes placed arbitrarily within the fluid. This gives several advantages in comparison to mesh-based methods when simulating nonlinear flow phenomena. The method uses discrete approximations to interpolation integrals to transform differential equations of fluid dynamics into particle summations. More complete reviews on standard SPH can be found at [3] and [4].

The SPH method is capable of dealing with problems involving large deformation such as free-surface flows, deformable boundaries, moving interfaces, wave propagation and solid simulation [5], [6]. However, a short period of physical time for these applications requires a large computational time when running on a single central processing unit (CPU) due to the large number of interactions for each particle at each timestep. This has hindered the development of SPH and its use by industry for real problems. Hence, with the objective of performing simulations that are industrially relevant, the ability to perform computations involving millions of particles is essential. However, this is only possible if some form of hardware acceleration is employed.

With present technologies, there are two main options for implementing hardware acceleration for CFD calculations: (i) using high-performance computing (HPC) on supercomputers consisting of thousands of CPU cores, or (ii) using the novel computing architectures such as Graphics Processing Units (GPUs) borrowed from the computer games industry. GPUs are designed to treat large data flows and to render pixels at a several tens of frames per second. From a computational point of view they are highly efficient thanks to their multi-threading capability. Due to the inexorable development of the video games market and multimedia, their computing power with streaming multi-processor technology has increased much faster than CPUs.

Thus, GPUs appear to be an accessible alternative to accelerate SPH models using a powerful parallel programming model where the graphics cards are used as the execution devices. Their performance can be compared with large cluster machines. Another important advantage is the cost and ease-of-maintenance of GPUs in comparison with large multi-core HPC systems.

The capability of GPUs to simulate SPH was demonstrated by the pioneering work of Harada [7]. Previously, only parts of the SPH scheme had been implemented on the GPU device, but in [7] the entire SPH computation was performed on the GPU. In that paper, the acceleration of SPH achieved was satisfactory where 60,000 particles were simulated in real time. When conducting tests involving 260,000 particles they obtained speedups of over 28 using a GPU compared to a CPU. The method proposed was implemented using a GeForce 8800GTX GPU card and developed before the appearance of the nVidia Compute Unified Device Architecture (CUDA). It is worth noting that CUDA is both a programming environment and language for parallel computing specifically for nVidia GPUs. Thus Harada's work represents a significant advance even when most of its limitations can now be addressed using the advanced GPU programming features introduced in the latest versions of CUDA. More recent work using GPUs for SPH can be found in [8], where the authors computed free-surface flows in coastal environments using a GeForce GTX280. In the work by Hérault and co-workers [8], [9] the speed-up achieved was on the order of 60 for a calculation involving more than 600,000 particles. In the fields of other meshless methods, one of the most recent implementing GPU flow solvers was performed for vortex particle methods [10], where the new solver was almost 30 times faster than their single-core CPU code.

SPHysics is an SPH numerical model developed in a collaborative effort amongst researchers at the Johns Hopkins University (US), the University of Vigo (Spain) and the University of Manchester (UK) (http://www.sphysics.org). Written initially in FORTRAN, a complete description of the software is found in the SPHysics user's guide [11]. The SPHysics group has focused its research mainly on wave propagation and interaction with coastal structures, both in 2D [12]–[14] and 3D [15]–[18]. Although the method allows a fine description of the flow in the nearshore areas, its main drawback is its high computational cost, so that the model cannot be efficiently applied over large domains. Hence, hardware acceleration must play an integral role in the development and application of SPH, and GPUs represent an accessible route for this objective. As a result the combined CPU-GPU code named DualSPHysics has been developed starting from the SPH features implemented in the FORTRAN SPHysics code. DualSPHysics was designed from the outset to use SPH for real engineering problems with software that can be run on either CPUs or GPUs. This DualSPHysics package can be freely downloaded from www.dual.sphysics.org and different applications can be viewed at http://www.vimeo.com/dualsphysics/videos.

In this paper, the solver is presented describing the main performance optimization techniques to implement SPH models using the GPU architecture. The GPU code will be shown to achieve up to two orders of magnitude speed-up compared to the CPU code. In addition, the numerical results will be validated with experimental data in order to show how the technique combines the accuracy of the CPU model presented in previous works with the efficiency of GPU programming.

## Methods

The description of the SPH formulation is beyond the aim of this paper; for a complete review about the main features of this technique the reader is referred to [3], [4], [13], [19], [20]. Here, we will provide a brief description of the method for solving the governing equations expressing conservation of mass and momentum. In the SPH formalism, the fluid domain is represented by a set of points (particles) scattered in a non-uniform arrangement which is modified each time step according to the governing dynamics. Thus, the physical properties of particles (mass, density, pressure, position, velocity) can change throughout the simulation due to the interaction of neighbouring points. This interaction depends on a weighting function, herein referred to as the smoothing kernel. These smoothing kernels must obey several key properties [19], namely, positivity inside a defined zone of interaction, compact support (i.e zero value outside that zone), normalization (partition of unity) and monotonically decreasing with distance. For most of the kernels, the weighting function vanishes for inter-particle distances greater than 2 h. Although, there is a wide variety of possible weighting functions (see [20] for a complete description), all the calculations shown in the present manuscript were carried out with a quintic (Wendland) kernel [21], [22].

### SPH Form of the Governing Equations

The momentum conservation proposed by [19] has been used to determine the acceleration of a particle (a) as the result of the particle interaction with its neighbours (particles b):
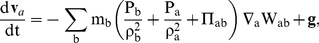
(1)where, **v** is velocity, P is pressure, ρ density, m mass, **g** = (0,0,−9.81) ms^−2^ the gravitational acceleration and W_ab_ the kernel function that depends on the distance between particle a and b.

Π_ab_ is the viscous term according to the artificial viscosity proposed in [19]:
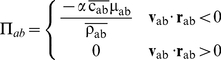
(2)with 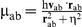; where **r**_ab =_ **r**_a_−**r**_b_, **v**_ab =_ **v**_a_−**v**_b_; being **r**_a_ and **v**_a_ the position and the velocity corresponding to particle a; 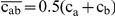
is the average speed of sound, 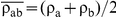 the mean density, η^2^ = 0.01 h^2^, and α = 0.01 a parameter according to [14], [15].

Alternative viscosity treatments have been considered in the literature; [23] proposed the laminar viscosity to solve problems involving low Reynolds number flows; [13] adapted the Sub-Particle Scale (SPS) approach to weakly compressible SPH; [24] proposed a different approach where viscosity depended on vorticity. Finally, [25] presented an overview on numerical modelling of complex turbulent free surface flows within the SPH context. Evidently, there are viscosity models in SPH that are more sophisticated than the artificial viscosity approach of equation (2). For free-surface flows, Monaghan and Kajtar [26] note that the parameter α in the artificial viscosity model can be related to the Reynolds number in the following manner 
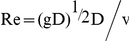
 where D is a characteristic water depth and 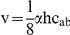 for a Wendland kernel.

The GPU scheme developed here for millions of particles allows the investigation of the global effect of implementing different viscosity models. However, their implementation and assessment of their accuracy is not the focus of this study, and hence artificial viscosity is sufficient for the simulations presented herein.

The mass of each particle is constant, so that changes in fluid density are computed by solving the conservation of mass or continuity equation in SPH form [19]:
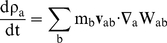
(3)The equations are closed by using Tait's equation of state to relate pressure to density [27], [28]:
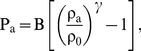
(4)where γ = 7 and, 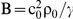 being ρ_0_ = 1000 kg m^−3^ the reference density and 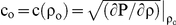 the speed of sound at the reference density.

In SPH schemes where pressure depends on density through an equation of state, the formulations are referred to as Weakly Compressible SPH (WCSPH). Alternatively, other authors have considered incompressible formulations solving a pressure-Poisson equation giving rise to strictly incompressible SPH (ISPH) methods. Numerous authors [29], [30], [31] have compared both methods and generally obtained improved pressure fields with the incompressible approach. Other authors [32], however, concluded that WCSPH performs at least as well as ISPH and in some respects even better. In terms of efficiency, WCSPH does not solve the Poisson equation which is computationally expensive; however, ISPH generally produces a pressure field with reduced pressure fluctuations so larger time steps are possible with ISPH. Overall, the efficiency of both methods is similar and WCSPH is adopted in the present study. It should be noted for the WCSPH approach, that the speed of sound must be slowed artificially to run simulations in reasonable times since the time step derived from the Courant condition is too small when realistic speeds of sound are used. Thus, following [33], the speed of sound must be at least ten times faster than the maximum fluid velocity to keep density variations within acceptable levels of less than 1%.

### Density Filters and XSPH correction

In WCSPH simulations, unphysical oscillations can be observed in the pressure field which is caused by the stiff equation of state (4) and inaccuracies in the kernel summation procedure itself (1,3). A straightforward and computationally inexpensive method to smooth these pressure oscillations is to perform a filter over the density of the particles and to re-assign a new density to each particle as done in [13], [34] following [35], [36].

The Shepard filter [13], [33] is, possibly, the simplest correction to the density field. In the present work, the filter is applied every N_f_ = 30 time steps as described in [4], although different values of N_f_ can be considered [34].

For the velocity field, each particle is moved according to the velocity in its neighbourhood, using the XSPH variant [37]. The parameter ε = 0.5 was chosen following previous research [4], [11], [12], [14], [15], [16], [17] as it prevents particle penetration as stated in [37]. In general, the influence of XSPH is limited when dealing with gravity dominated problems, especially for variables such as water height that correspond to the mean movement of large volumes of fluid. Other variables, e.g. pressure near boundaries, are more sensitive to the actual value of ε. Local pressure depends on density according to Eq. 4 and density, itself, depends on the distance between particles according to Eq. 3. When ε = 0, the velocity of each particle is not smoothed using the XSPH correction, so it is possible that this is fluctuating unphysically in comparison with the surrounding velocity field of its neighbours. Hence, a single particle can approach a boundary at high velocity, giving rise to an unrealistic increase in density and, hence, pressure. For ε values on the order of 0.5, the velocity of every particle is influenced by the movement of its neighbourhood and the possibility of a single particle moving much faster than its neighbours is smaller, reducing the appearance of spikes in pressure.

### Time stepping

As mentioned above, the physical quantities (velocity, density, position and density) change each time step due to particle interactions. In SPH time integration schemes must be at least second order since the particle represent computation points moving according to the governing dynamics. In particular, a Verlet [4], [13], [14], [16], [38], [39] algorithm will be used in the present work.

A time-step control which depends on the CFL (Courant-Friedrich-Levy) condition, the forcing terms and the viscous diffusion term [37] will be considered. The variable time step Δ*t* will be calculated according to [33].

### Boundary conditions

In this work, boundary particles are used to create a repulsive force to prevent fluid particles from penetrating the limits of the domain or solid objects. Herein, we will use ‘dynamic’ boundary conditions previously employed in [4], [11], [12], [14], [15], [16], [39]. These boundary particles satisfy the same equations of continuity and state as the fluid particles, but their positions remain unchanged or are externally imposed. This type of boundary condition is easy to implement due to its computational simplicity where the interactions fluid-boundary can be calculated inside the same loops as fluid particles. For complex boundaries, the choice of this boundary condition is justified due to the difficulty to calculate normal and tangent vectors for arbitrary geometries [33].

### Implementation on CPU and GPU

The SPH scheme presented in the previous section is implemented in the DualSPHysics code. The new code was developed starting from the former Fortran SPHysics model and implemented using both the C++ and CUDA programming languages. The code can then be executed either on the CPU or on the GPU since all computations have been implemented both in C++ for CPU simulations and in CUDA for the GPU simulations. The philosophy underlying the development of DualSPHysics is that most of the source code is common to CPU and GPU which makes debugging straightforward as well as the code maintenance and new extensions. This allows the code to be run on workstations without a CUDA-enabled GPU, using only the CPU implementation. On the other hand, the resulting codes should be necessarily different since code developers have considered efficient approaches for every processing unit. As explained below, the same programming strategy can be efficient on a CPU but inefficient on a GPU (or vice versa). Thus, comparisons between the performances of both approaches are more reliable since appropriate optimisations have been considered for every case.

**Figure 1:**
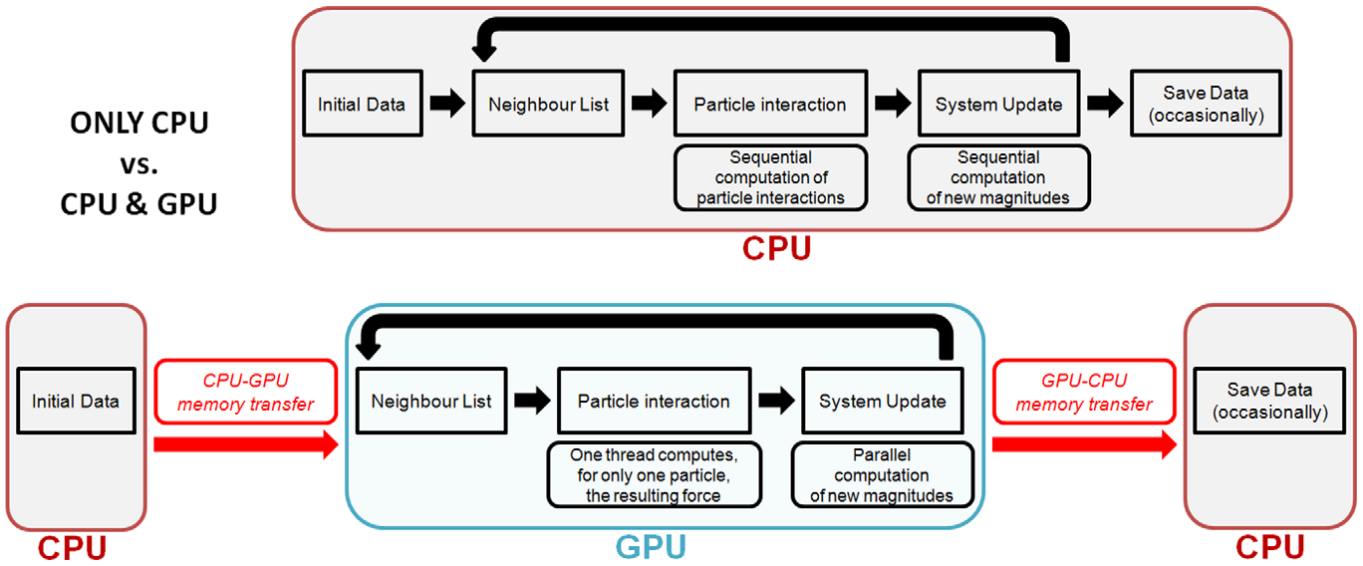
Flow diagram showing the differences of the CPU and GPU implementations. Implementation only based on CPU is represented in the upper part and CPU/GPU implementation is shown in the lower part of the figure.

The code is organised in three main stages that are repeated each time step: (1) creating a neighbour list; (2) computing particle interactions for momentum and continuity conservation equations; and (3) time integration referred to as a system update herein. Figure 1 shows the scheme of the different implementations on CPU and GPU.

### CPU Implementation

The CPU implementation is shown in the upper panel of Figure 1. The iterative process of the SPH implementation is shown in the figure using the long thick black arrow connecting system update and neighbour list.

During the first step the neighbour list is generated. The cell-linked list described in [40] is implemented. This process can be divided into different operations: (i) domain division into square cells of side 2h, (or the size of the kernel domain) following [41], (ii) determining the cell to which each particle belongs, (iii) reordering the particles according to the cells, (iv) ordering all arrays with data associated to each particle and, finally, (v) generating an array with the position index of the first particle of each cell. This linked list is described in more detail in [40]. This means that a list of neighbours for each particle is not created, only a list of particles is generated. Thus, for a particle located inside a cell, only the interactions with the particles of neighbouring cells need to be considered. In this way the number of calculations per time step is reduced from N2 operations (being N the number of particles) to approximately N·logN or less.

Secondly, the force computation is performed so that all particle interactions are solved according to the SPH equations. Each particle interacts with all neighbouring particles located at a distance less than 2 h. Only particles inside the same cell and adjacent cells are candidates to be neighbours. Kernel symmetry, and hence kernel gradient asymmetry, avoids unnecessary repetition of particle interactions leading to a minor improvement in performance. When the force interaction of one particle with a neighbour is calculated, the force of the neighbouring particle on the first one is known since they have the same magnitude but opposite direction. Thus, the number of adjacent cells to search for neighbours can be reduced if the symmetry in the particle interaction is considered, which reduces the computational time [11], [40].

Finally, the time step is computed and the quantities at step n+1 are calculated from the quantities that are already known at step n.

### GPU Implementation

Computational runtime increases dramatically with the number of particles in the SPH simulations. Hence, parallelisation methods are essential to run simulations with a huge number of particles in a reasonable execution time. GPUs constitute a suitable hardware for scientific tasks where mathematical calculations are carried out using large sets of data. Consequently, DualSPHysics merges the accuracy, stability and reliability shown by the former SPHysics code with the performance enhancement available from GPUs and CUDA. The work presented in [42] introduced the framework to implement SPH codes using the best techniques and performance optimizations on GPU. That work focused on identifying suitable algorithms for efficient parallelization since a proper and full use of all the capabilities of the GPU architecture is not straightforward. As an initial step, the implementation focused on solving the particle interactions on a GPU using CUDA and the next step was the implementation of the neighbour list and the time integration in order to develop an entire GPU-SPH model. Different neighbour lists were analysed in [40] for the SPHysics code. Apart from a non-negligible improvement in the performance of the model, the work also showed that computing particle interactions (step 2 mentioned above) is the most expensive SPH procedure in terms of computational runtime. This influences the development of a GPU code.

In a first approach, it is possible to keep the other two steps (neighbour list and system update) on the CPU. However, this is less efficient since particle data and neighbour list information must be transferred between both processing units each time step, which consumes hundreds of clock cycles. The most efficient option is keeping all data in the memory of the GPU where all processes are parallelised. Only output data requires transfer from GPU to CPU. This process is rarely carried out (one out of one hundred time steps at most) and only represents a low percentage of the total runtime.

A preliminary version of the DualSPHysics code with a total GPU implementation was presented in [43]. The lower panel of Figure 1 represents the GPU implementation. Initially, data is allocated on CPU, so there is a single memory transfer (from CPU to GPU). In all subsequent calculations, the three main steps are then performed on the GPU device. All the sequential tasks and operations that involve a loop over all particles are performed using the parallel architecture of the GPU cores. To save (or output) data, a new memory transfer is needed (from GPU to CPU). If saving data is not required all particle information remains on the GPU memory and is only updated each time step.

**Figure 2:**
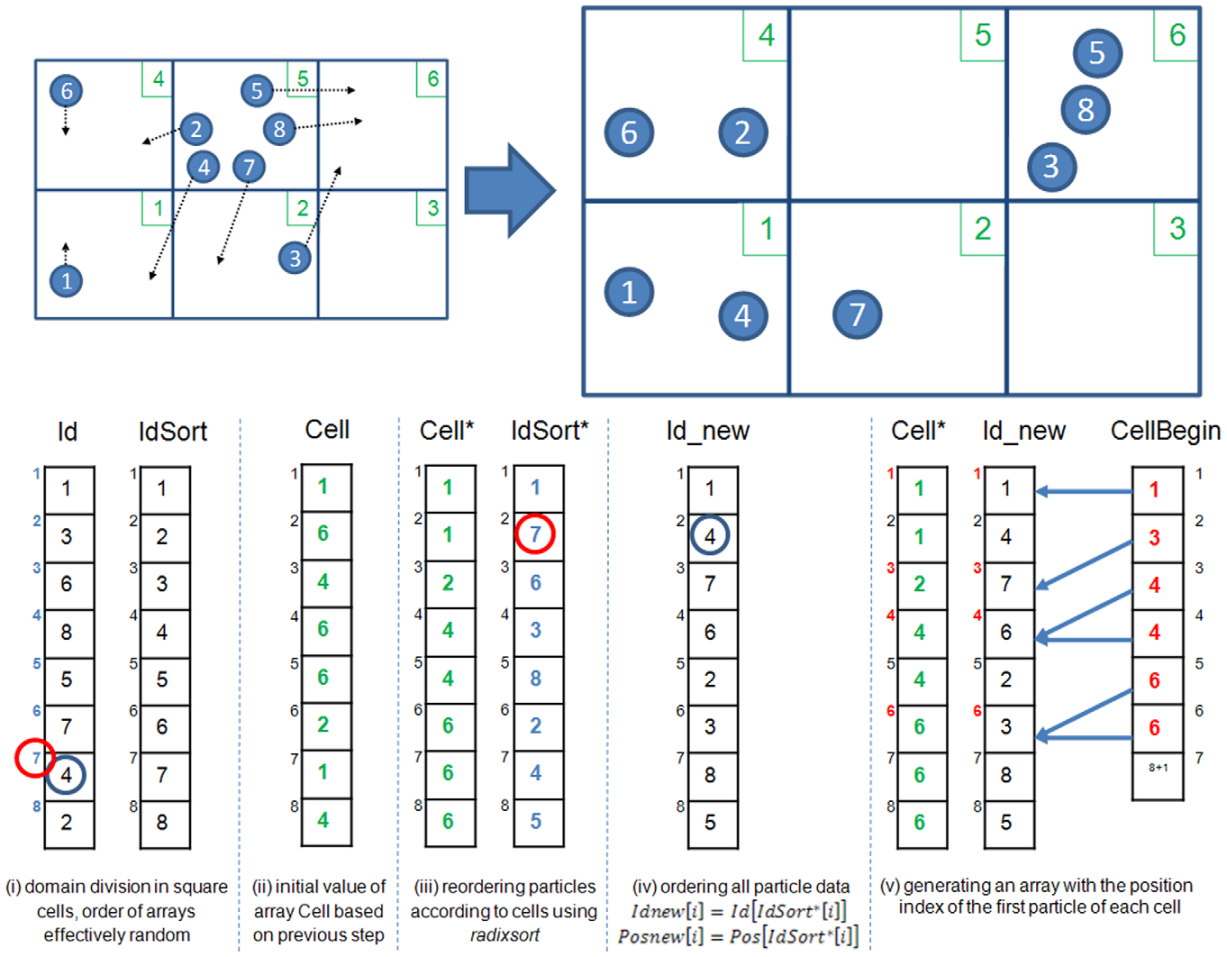
Example of the Neighbour list procedure.

The neighbour list creation follows the procedure used on a CPU, but with several differences. Reordering the particles according to the cells they belong is computed using the optimised *radixsort* algorithm provided by CUDA. Figure 2 shows a simplified schematic diagram of the method used to generate an array of particle labels ordered according to cells and an array with the position index of the first particle in each cell. Four separate arrays are used: Id, Cell, IdSort and CellBegin with a superscript * denoting sorted arrays. The array Id (array of particle labels) is the starting point with particles randomly located in the domain, where the order of this array corresponds to the list of particles inherited from the previous timestep. The neighbour list is created according to the following steps:

- Particles are stored according to the cells, so the array IdSort is created.
- The array Cell is also created where an entry gives the cell to which the particle of the same index in Id belongs, e.g. Id(2)  =  particle 3 which is located in Cell 6 hence Cell(2)  = 6. Cell labels are depicted in green colour in Figure 2.
- Using radixsort, array Cell is reordered following the order of the six cells and Cell* (reordered Cell) is used to reorder IdSort according to the cells the particles belong.
- Once IdSort* is generated, all the arrays with particle information (Id, Position, Velocity, Density...) are ordered giving rise to the new arrays (Id_new, Pos_new, Vel_new, Dens_new...) considering that Id_new [i]  =  Id [IdSort* [i] ]. For example, Id_new [2]  =  Id [IdSort* [2] ]  =  Id [7]  = 4, in Figure 2 a blue circle marks the particle 4 and a red circle marks the 7th position.
- Finally, CellBegin is created with the indexes (position in data arrays) of the first particle of each cell. Indexes have been written in red colour in Figure 2. For example the first particle of the cell number 2 is the particle 7, whose position index is 3 in all particle property arrays, so the second value of CellBegin, which corresponds to cell number 2, will be 3. In this way, the amount of particles in the cell k will be CellBegin[k+1]-CellBegin[k].

**Figure 3:**
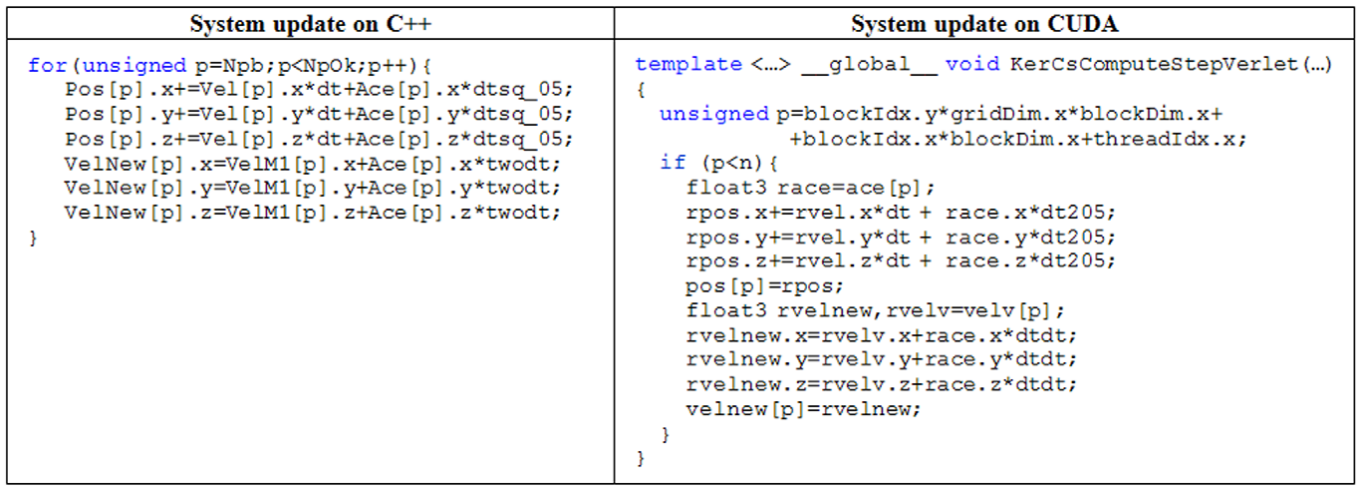
Pseudocode of the System update procedure implemented on CPU and GPU.

The system update associated with time integration can be parallelised easily on a GPU. Example pseudocode is shown in Figure 3 where similarities between the CPU and GPU versions are clearly evident and demonstrates the advantages of a using C++ and CUDA when developing code. The new time step is computed according to [11] where the maximum and minimum values of different variables (force, velocity and sound speed) are calculated. This calculation is optimised using the *reduction* algorithm (also provided by CUDA). Reduction algorithm allows obtaining the maximum or minimum values of a huge data set taking advantage of the parallel programming in GPUs.

As mentioned above, the particle interactions of the force computation are a key process that must be implemented in parallel in order to improve the performance of the model. The use of the shared memory of the GPU was analysed to reduce the access to the global memory of the GPU. However, when the SPH code is implemented entirely on the GPU, this technique is not viable. For example, when the number of particles is large, the size of shared memory is not enough to allocate the properties of all the particles belonging to the same cell. Particle interactions can be implemented on the GPU for only one particle using one execution thread to compute the force resulting from the interaction with all its neighbours. This technique presents several limitations mainly due to the Lagrangian nature of the method. On the one hand, the workload of threads inside one block is not balanced since particles can have different numbers of neighbours. On the other hand, code divergence can appear since when the possible neighbours of a particle are evaluated, some of them are definite neighbours (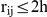) and the force computation is performed while other particles are not neighbours (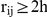) and no computation is performed. Note that according to the link list described in [11], [40] the potential neighbours are all particles located in adjacent cells. Nevertheless, only those particles at distances less than 2 h from the target particle are real neighbours.

**Figure 4:**
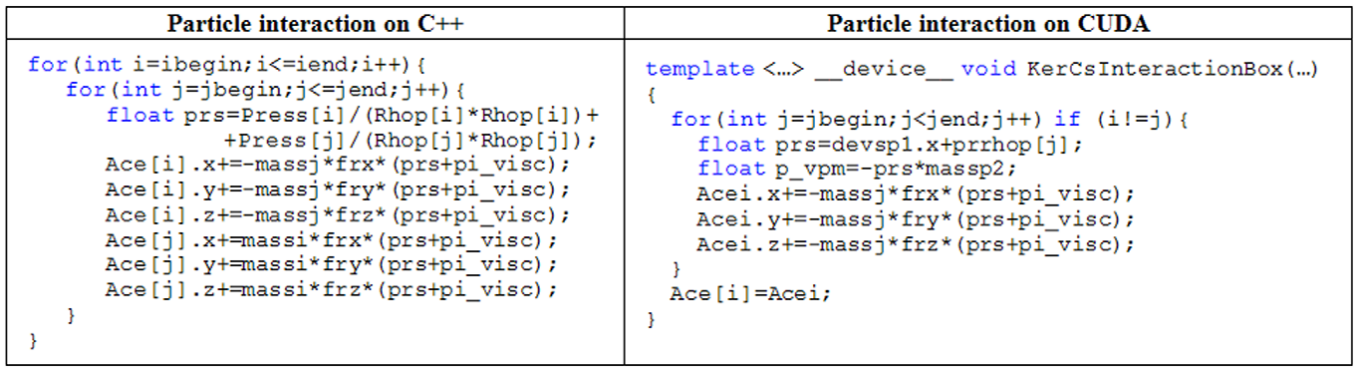
Pseudocode of the Particle interaction procedure implemented on CPU and GPU.

An important difference here from the CPU part of the DualSPHysics code is that the symmetry of the particle interaction cannot be applied on a GPU implementation since each thread is responsible for the interaction between a target particle and its neighbours, so that each thread must be the only one that computes the forces exerted on that particle. The access to the global memory of the device is irregular because there is no way to organise the data to get a coalescent access for all the particles. If a second thread tried to modify those forces, as could occur when considering particle kernel symmetry, it would generate erroneous results when both threads accessed simultaneously the same variable. This effect can be mitigated by synchronising the threads but it would dramatically reduce the performance of the model. An example of the similarity of the C++ and CUDA codes for this illustrative point is shown in Figure 4.

## Results

In this section, we investigate the performance of the DualSPHysics code with a standard free-surface benchmark test for SPH flows, a dam-break experiment, in order to demonstrate the reliability, capability, accuracy and efficiency of the CPU-GPU solver. The test case was simulated to analyse the agreement between numerical and experimental data examining the effect of the number of particles.

### The experiment for validation

The experiment described in [44] consists of a dam break flow impacting with an obstacle. This experiment is considered a valuable benchmark for the SPH free-surface flow community (http://wiki.manchester.ac.uk/spheric/index.php/Test2).

**Figure 5:**
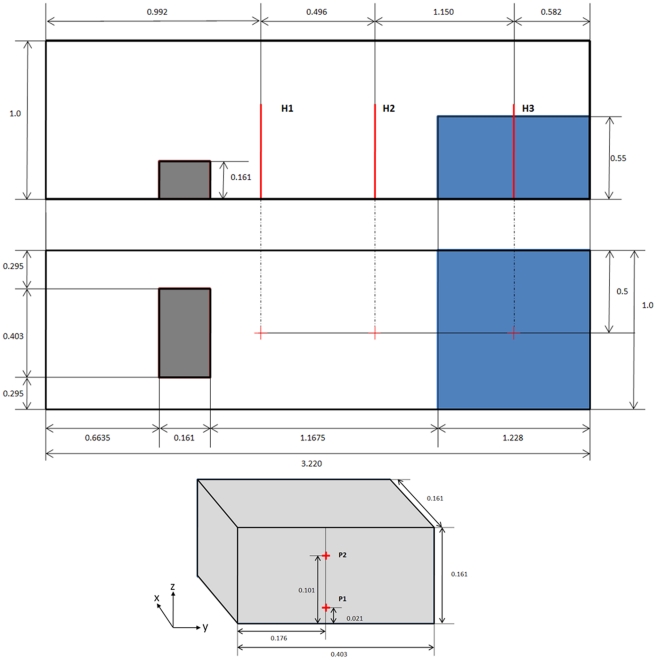
Experimental configuration of the [**44**] experiment and measeurement positions for the experimental data: Side view, top view and location of pressure sensors.

The experimental configuration is depicted in Figure 5. The tank is 3.22 m long, 1 m wide and 1 m tall. The volume of water is initially confined at one end of the tank in a volume 1.228 m long, 1 m wide and 0.55 m tall and is released instantaneously at the start of the simulation. With the removal of the retaining wall, the fluid floods the dry bed of the tank due to gravity.

The experiment [44] provides water heights and pressure measurements at different locations. Three vertical height probes (H1, H2, and H3) were used to determine the water height during the experiment. H3 was placed in the position of the water reservoir and the other two were placed at different locations along the tank (Figure 5). Pressures exerted on the obstacle initially facing towards the water were also sampled to detect the water impacts.

### Numerical results

**Figure 6:**
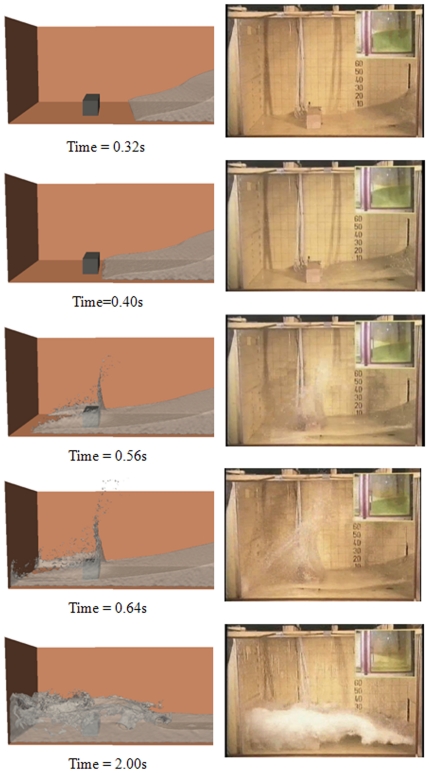
Different instants of the SPH simulation for the testcase. Right snapshots correspond to figures from [44].

The SPH simulations were carried out using the DualSPHysics code. Three simulations with a different number of particles are analysed: (i) 10,000 particles (h = 7.5×10^−2^ m), (ii) 100,000 particles (h = 3.075×10^−2^ m) and (iii) one million particles (h = 1.35×10^−2^ m). Figure 6 shows different instants of the SPH simulation using one million particles. The fluid simulation performed by DualSPHysics (left panel) is close to the experiment (right panel). The first instant (t = 0.32 s) reproduces the dam release. Just after t = 0.40 s, water hits the obstacle. Then, the fluid splits with an upward-moving jet formed after impact while the rest of the fluid surrounds the obstacle (t = 0.56 s and t = 0.64 s). The last frame (t = 2.0 s) shows a splash due to the reflected wave generated after hitting the left wall.

### Water depth comparison

Numerical depth probes were computed to compare with the experimental measurements. These numerical probes constitute a set of points where the mass is computed as an interpolation of the mass of the neighbouring fluid particles. Points do not correspond to the physical positions of particles, thus the interpolated mass at the location p is calculated following:
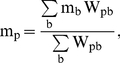
(5)where b denotes all the fluid neighbouring particles around point p, m_b_ is the mass of each fluid particle b and W_pb_ is the kernel function calculated in terms of the distance between the positions of the fluid particle and the node p.

This procedure, which has been used previously by the authors [12], [15] is based upon the fact that there is an abrupt change in mass at the free surface. Thus, a reference value of mass, 0.5 m_b_, was chosen to determine the maximum height. Note that all particles b have exactly the same mass and hence the position of the free surface corresponds to the point where the calculated mass, m_p_, equals 0.5 m_b_ . Therefore, given a particular location in space, the highest elevation where the interpolated mass, m_p_, is higher than the reference mass is considered to be the water height at that location. Water heights were computed at different instants and compared with the experimental data.

**Figure 7:**
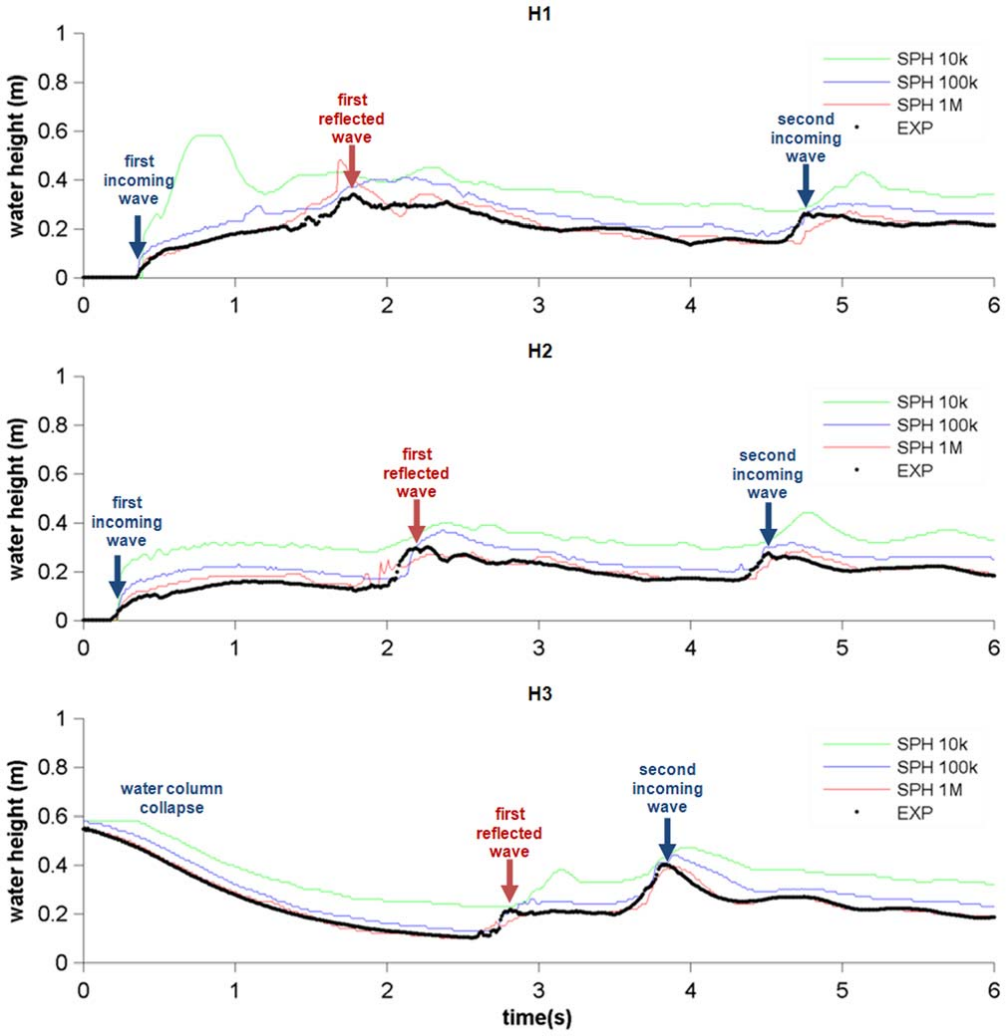
Experimental and numerical water heights measured at the three probes.

Different SPH simulations were carried out using DualSPHysics with different resolutions (numbers of particles). Figure 7 summarises the experimental and numerical water heights calculated at the three probes located before the obstacle (H1, H2 and H3). The black line corresponds to the experimental water height data, the green line corresponds to the simulation using 10,000 particles, the blue line corresponds to the simulation with 100,000 particles and the red line corresponds to one million particles.

The water column collapse is observed during the first two seconds. This dam break is clearly shown by the probe at H3, where the water level decreases during this period and by the other probes where the water arrives sequentially (first at H2 and then at H1). After 1.75 s the reflected waterwave moves to the right after hitting the left wall. The reflected wave hits the right wall and a second incoming wave hits the obstacle for a second time on the right side (a second maximum in the water level is detected by H3 at 3.8 s, later by H2 at 4.6 s and by H1 at 4.8 s). The SPH results reproduce properly the dam evolution observed in the experiment. However, some differences are now addressed between numerical and experimental results. During the second incoming wave, the numerical signal is slightly delayed in comparison with the experimental one. This difference increases when the number of particles decreases. The same validation case was previously used in [45], showing similar differences. The authors related these deviations to the interaction with the boundary during the impact on the back and front walls, concluding that SPH could be overestimating the boundary effect on the flow. The treatment of boundary conditions is still an open field in SPH and new research should be conducted ([46]).

The agreement between experimental wave heights and SPH results can be quantified considering two statistical parameters; amplitude A_F_ and phase P_F_:
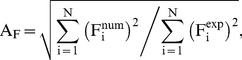
(6)

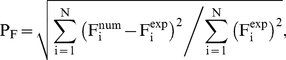
(7)where F_i_ is the magnitude to be analysed (water elevation in this case), num refers to numerical values, exp to experimental values and N is the number of samples. These parameters were previously used in [4], [14] to determine the accuracy of the SPHysics model.

The values of these parameteres are presented in Table 1. A perfect agreement between the signals would result in A_F_ →1 and P_F_ →0. All amplitude values (A_F_) shown in Table 1 are close to unity showing the good agreement between the SPH simulation and the experiment. In addition, the best results are obtained using the highest number of particles. This implies the convergence of the numerical model when increasing the resolution. Furthermore, the delay between the numerical and the experimental signals observed in Fig. 7 can also be studied in terms of the phase parameter, whose value is never close to zero. Nevertheless, P_F_ is observed to decrease when increasing the resolution showing the convergence of the model.

**Table 1:** Statistical comparison between the positions of the free-surface measured in the experiment (*exp*) and calculated by DualSPHysics (*num*).

Height Gauge	Number of particles	Amplitude	Phase
*H1*	*10* *k*	1.71	1.49
	*100* *k*	1.27	1.04
	***1M***	**1.04**	**0.80**
*H2*	*10* *k*	1.71	1.51
	*100* *k*	1.28	1.07
	***1M***	**1.02**	**0.81**
*H3*	*10* *k*	1.46	1.17
	*100* *k*	1.18	0.88
	***1M***	**1.00**	**0.68**

In bold are shown the best agreements.

### Pressure comparison

Pressure was also measured experimentally. Different pressure sensors (Figure 5) were used to collect the experimental pressure on the obstacle. The pressure on the front side of the obstacle (P1 and P2) was computed by DualSPHysics to analyse the validity of the model to predict the forces exerted by the fluid on the structure. Numerical pressures were computed at the positions where the experimental sensors were located.

As mentioned above, the dynamic boundary particles evolve according to the same conservation equations and state equation as the fluid ones. This allows the density and pressure to be computed for these particles at each time step. The interpolation described in Eq. 5 was used to calculate pressures at the positions where experimental sensors were located:
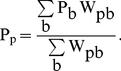
(8)Note that only boundary particles were used to calculate pressure. Thus, b denotes all the boundary neighbouring particles around sensor point p, P_b_ is the pressure of each boundary particle b and W_pb_ is the kernel function calculated in terms of the distance between the positions of the boundary b and the node p.

**Figure 8:**
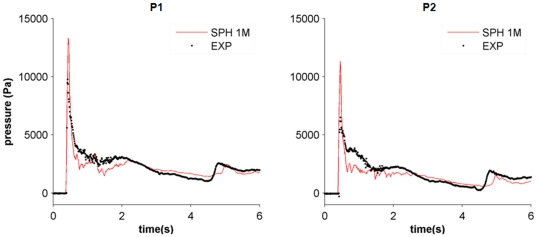
Experimental and numerical pressures using one million particles.

The comparison between experimental and numerical pressures is shown in Figure 8. The numerical values correspond to the simulation with one million particles since this resolution provided the most accurate results when computing water heights. A close agreement between both signals can be observed for this resolution. In Figure 8, the maximum experimental and numerical peaks, which correspond to the main water impact on the obstacle, coincide in time although their magnitude is different. This behaviour is consistent with other SPH simulations (e.g. [18]). Even, the presence of a secondary peak at approximately 4.5 seconds is also detected by the numerical simulation, although it is a slightly delayed with respect to the experimental one. Table 2 shows the differences in phase and amplitude between experimental and numerical results calculated with Eqs. (6) and (7). A similar comparison was carried out in [30], where the accuracy of WCSPH and ISPH approaches was analysed.

**Table 2:** Statistical comparison between the pressure values measured in the experiment (*exp*) and calculated by DualSPHysics (*num*).

Pressure Gauge	Number of particles	Amplitude	Phase
*P1*	*1* *M*	1.07	0.46
*P2*	*1* *M*	1.04	0.56

Finally, all the results presented in this validation were obtained using single precision, which was enough to reproduce accurately the water elevation and pressure measured in the experiments. A study using double precision can also be carried out since the latest CUDA-enabled GPU cards present improved support for double precision. The preliminary results here show that the differences with single precision calculations are smaller than the uncertainties in experimental results, thus using double precision is not necessary.

### Efficiency using GPU

Once the code has been validated and the accuracy of the numerical results has been assessed, the efficiency of using a GPU can be analysed. The test case described in the previous section is simulated both on a CPU and a GPU to analyse the performance of DualSPHysics code.

The CPU implementation on C++ is carried out on different CPUs (Intel® Core™ i7 940 at 2.93, Intel Xeon X5500 at 2.67GHz and Intel Xeon E5620 at 2.4GHz). The GPU element of the DualSPHysics code is run on four different cards: GTX 260, TESLA M1060, GTX 285 and GTX480 (see Table 3 for general specifications).

**Table 3:** General specifications of the different GPUs.

	Number of cores	Processor clock	Memory space	Maximum number of particles using *DualSPHysics*
**GTX 260**	192	1.24 GHz	0.875 GB	4.75 million
**TESLA M1060**	240	1.36 GHz	4 GB	21.72 million
**GTX 285**	240	1.48 GHz	1 GB	5.43 million
**GTX 480**	480	1.40 GHz	1.5 GB	8.14 million

**Figure 9:**
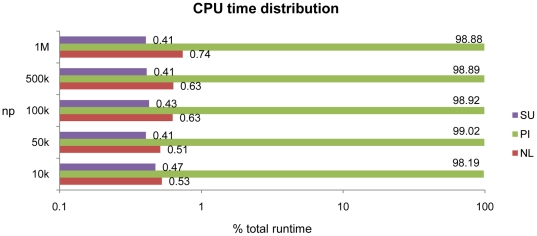
Computational runtime distribution on CPU (Intel i7).

Firstly, the profiling of the execution time of the SPH processes in DualSPHysics is analysed to justify the implementation approach described above. Thus, Figure 9 represents the CPU runtime distribution of the three main SPH steps; neighbour list creation (NL), particle interaction (PI) and system update (SU). Considering different numbers of particles (np), the particle interaction always takes 98–99% of the total computational time. This was the justification for applying GPU parallelism to this process considered initially to accelerate the code. NL and SU take 0.6–0.8% and 0.4% respectively, however keeping these steps on the CPU is less efficient due to the cost of data transfer between CPU and GPU at each time step. In this way, the three processes are parallelised and the whole SPH simulation is implemented on the GPU.

**Figure 10:**
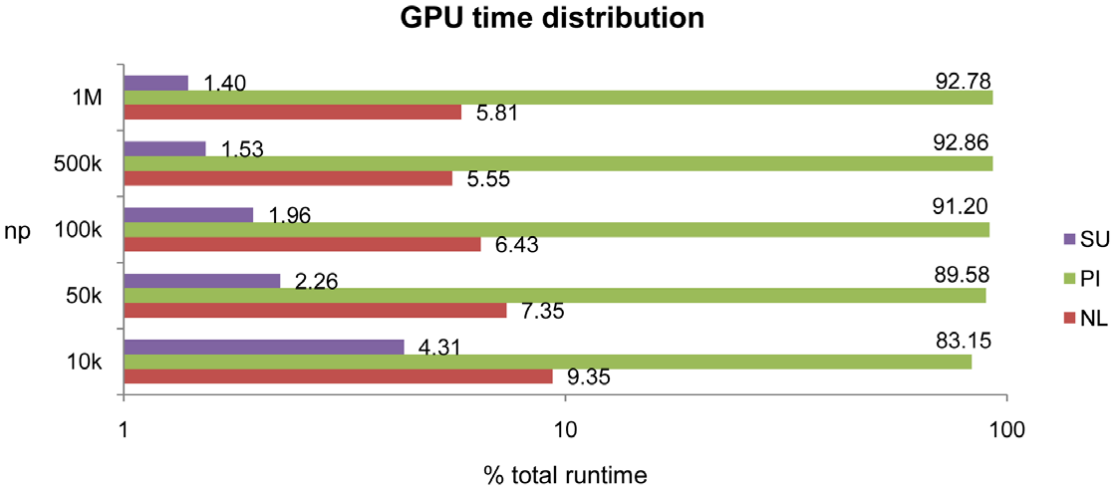
Computational runtime distribution on GPU (Tesla M1060).

Figure 10 shows the new time distribution once the NL, PI and SU are performed entirely on the GPU device. Particle interaction times range from 81% (low resolution) to 92% (high resolution) of the total runtime. The percentage of computational time used for NL and SU is larger than observed for CPU calculations, although it decreases when increasing the resolution.

**Figure 11:**
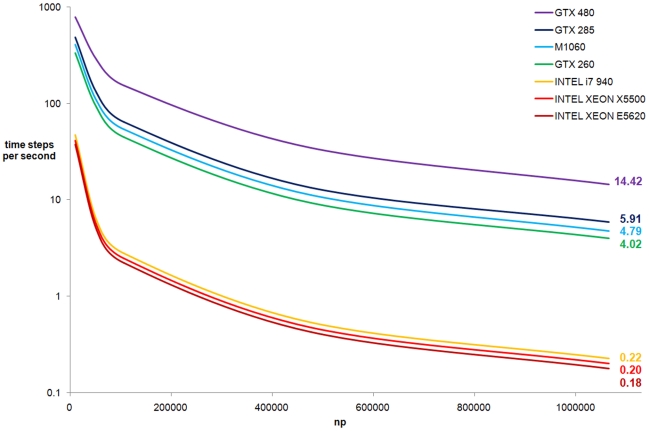
Performance of DualSPHysics code.

The performance of different simulations of the same testcase is presented for 3.0 seconds of physical time. The performance was analysed for different resolutions by running calculations with different numbers of particles. Figure 11 shows the number of time steps computed per second calculated for different devices (i.e. CPUs and GPUs) using different programming languages. For the sake of clarity the scale of the Y-axis (time steps computed per second) is logarithmic.

For one million particles, the performance of a CPU ranges from 0.18 to 0.22 time steps per second, while 4.02–14.42 time steps per second can be computed with a GPUs. The whole computation takes more than 5 days on the Intel® Core™ i7 (best CPU result) and less than 2 hours on the GTX 480 (best GPU result), resulting in a speedup of 64 (experience has shown with other similar test cases not reported here that this speedup can be even higher). Although the TESLA M1060 card presents some of the highest computational specifications in terms of memory (4 GB), the GTX 480 card provides the best efficiency. The GTX 480 belongs to the new FERMI technology and presents the maximum number of cores of the GPUs used in this work (see Table 3). GTX 480 is 2.4 times faster than the GTX 285 and 2.8 times faster than the TESLA card. Note that the obtained performance corresponds to the best approaches and optimised codes for CPU and GPU.

The results depicted in Figure 11 are computed without saving output data to the CPU. When a real simulation is studied, some information will be saved to analyse the numerical results. To analyse the cost of saving data, three hundred output files are saved in binary format during a simulation on the TESLA M1060 card representing three seconds of physical time. The time dedicated to save those data, at a sampling frequency of 100 Hz, only takes around 0.01% of the total simulation.

Finally, some authors [10] have pointed out the existence of differences in accuracy when using CPUs and GPUs, especially for simulations involving high Re numbers. In the present study both approaches have shown the same accuracy when compared with experimental results.

## Discussion

A CPU-GPU solver named DualSPHysics has been developed to deal with free-surface flow problems requiring high computational cost. The model was developed from the classical SPHysics FORTRAN code, inheriting the properties of stability and accuracy of its predecessor. The code can be run as either a CPU code or a GPU code depending on the availability of hardware. The model has been demonstrated to be both accurate and efficient when dealing with a gravity-dominated flow problem.

The code was validated using a dam break impacting with an obstacle. This experiment, which is a classical benchmark for the free-surface flow SPH community (http://wiki.manchester.ac.uk/spheric/index.php/Test2), provides water elevation and pressure data sampled at different locations. Simulations carried out for different resolutions showed a close agreement between numerical and experimental results. In addition, the numerical results were observed to converge to the experimental ones when increasing the resolution (the number of particles), both for free-surface elevations and pressures.

In terms of efficiency, we have demonstrated that simulations with a large number of particles can be simulated on a personal computer equipped with a CUDA-enabled GPU card taking advantage of the performance and memory space provided by the new GPU technology. This means that research can be conducted with available cheap technology for problems that previously required high-performance computing (HPC). The speedups obtained in this work reveal the possibility to study real-life engineering problems at a reasonable computational cost. For the validation case chosen here, the GPU parallel computing can accelerate serial SPH codes by almost two orders of magnitude, e.g. the FERMI card is 64 times more efficient than the best CPU single-core. Experience has shown that the speedup varies from one test to another with even greater speedup achievable than found here. The achieved performance can be compared to the large cluster machines, which are expensive and hard to maintain. For example, according to [47], where the authors simulated complex flows on the IBM supercomputer Blue Gene/L at Ecole Polytechnique Fédérale de Lausanne (EPFL–Switzerland), it would be necessary to use around 100 cores to equal the speedup achieved by only a single CUDA-enabled Fermi Card.

Finally, for simulations requiring several million particles the immediate future for GPU computing should focus upon multi GPU implementations, since the memory requirements are still a limitation for a single GPU. The SPHysics team has made significant advances in that direction but the efficiency of the communication between GPUs is still an open question.
